# Differentiating HER2-low and HER2-zero tumors with 21-gene multigene assay in 2,295 HR + HER2- breast cancer: a retrospective analysis

**DOI:** 10.1186/s13058-024-01911-9

**Published:** 2024-11-06

**Authors:** Yoonwon Kook, Young-jin Lee, Chihhao Chu, Ji Soo Jang, Seung Ho Baek, Soong June Bae, Yoon Jin Cha, Gyungyup Gong, Joon Jeong, Sae Byul Lee, Sung Gwe Ahn

**Affiliations:** 1 Department of Surgery, Gangnam Severance Hospital, Yonsei University College of Medicine, 712 Eonjuro, Gangnam-gu, Seoul, 06273 Republic of Korea; 2 Institute for Breast Cancer Precision Medicine, Yonsei University College of Medicine, Seoul, Republic of Korea; 3 Department of Surgery, Asan Medical Center, University of Ulsan College of Medicine, Seoul, Republic of Korea; 4 Department of Pathology, Gangnam Severance Hospital, Yonsei University College of Medicine, Seoul, Republic of Korea; 5 Department of Pathology, Asan Medical Center, University of Ulsan College of Medicine, Seoul, Republic of Korea

**Keywords:** Breast cancer, HER2-low, 21-gene multigene assay

## Abstract

**Background:**

HER2-positivity is an essential marker for therapeutic decisions, while HER2 expression is heterogenous. In recent years, there has been increasing recognition of a subgroup of breast cancer patients who have low levels of HER2 expression, also known as HER2-low because trastuzumab deruxtecan offers clinical benefit for patients with HER2-low metastatic breast cancer. Despite the growing interest in HER2-low breast cancer, there is limited research on how multigene assays can help differentiate between HER2-low and HER2-negative breast cancer. Among HR + HER2- breast cancer, we compared genomic characteristics between HER2-low and HER2-zero using the 21-gene assay.

**Methods:**

A retrospective review of clinical records was performed in 2,295 patients who underwent Oncotype DX^®^ test in two hospitals between 2013 and 2020. Patients were classified into two groups as the HER2-zero and HER2-low based on HER2 immunohistochemistry. In cases with HER2 2+, no amplification of HER2 gene was confirmed by silver in situ hybridization. High genomic risk was defined as cases with 21-gene recurrence score (RS) > 25. Multivariable binary logistic-regression analysis was performed.

**Results:**

Of these, 944 (41.1%) patients were assigned to the HER2-zero group, while 1351 (58.9%) patients were assigned to the HER2-low group. The average Recurrence Score (RS) was found to be 17.802 in the HER2-zero breast cancer group and 18.503 in the HER2-low group, respectively (p-value < 0.005). When comparing the proportion of high RS between the two groups, the HER2-zero group had a high RS rate of 12.4% (117 out of 944), while the HER2-low group had a high RS rate of 17.0% (230 out of 1351) (*p* = 0.002). The HER2 score identified by qRT-PCR was 8.912 in the HER2-zero group and 9.337 in the HER2-low group (*p* < 0.005). In multivariable analysis, HER2-low status was found to be an independent factor for high RS, with an odds ratio of 1.517 (1.172–1.964), independent of ER, PR, and Ki67. Within the subgroup of patients with invasive ductal carcinoma, the high RS rates were 19% in the HER2-low group and 14% in the HER2-zero group. However, when considering all patients, there were no significant differences observed in recurrence-free survival and overall survival between the HER2-low and HER2-zero groups.

**Conclusion:**

Within HR + HER2- breast cancer, HER2-low tumors are associated with high RS, especially for histologically invasive ductal carcinoma. A prognostic influence of HER2-low expression among HR + HER2- breast cancer remains as an area that requires further study.

## Introduction

Breast cancer is a heterogeneous disease with distinct subtypes, each characterized by unique clinical and molecular features [1, 2]. The most common subtype is hormone receptor positive (HR+) and HER2 negative (HER2-) [3, 4]. In the management of this subtype, treatment decisions are guided not only by TNM stage and clinicopathologic characteristics but also by the utilization of multigene assays, such as the 21-gene assay and the 70-gene signature, when appropriate [3–5]. 

The introduction of HER2-targeted therapy has significantly improved survival outcomes for patients with HER2 positive breast cancer [6–10]. Recently, the benefit has been extended to patients with low expression of HER2, especially by the development of antibody-drug conjugates mainly through a bystander effect [11–13]. As a result, there is increasing attention to better understand these HER2-low breast cancer on a clinical and molecular level [14]. 

The debate regarding whether HER2-low breast cancer should be regarded as a distinct subtype has been fueled by a series of studies that have yielded varying results [14–17]. These studies have been made to find an answer to this question; however, the evidence has not yet accumulated enough to provide conclusive support for either perspective.

Despite the increasing interest in HER2-low breast cancer, there is a paucity of research examining how multigene assays can aid in distinguishing between HER2-low and HER2-negative breast cancer. In this study, we sought to compare the Recurrence Score (RS) derived from the 21-gene assay between HER2-low and HER2-zero subgroups within ER + HER2- breast cancer.

## Methods

### Study population

This study adhered to the guidelines for reporting observational studies outlined by the Strengthening the Reporting of Observational Studies in Epidemiology initiative. A retrospective review was conducted on the clinical records of 2295 patients with HR + HER2- early breast cancer, who were treated at Gangnam Severance Hospital and Asan Medical Center between 2013 and 2020. Ethical approval was obtained from the institutional review boards of both hospitals, and informed consent was waived due to the retrospective nature of the study.

**Fig. 1:**
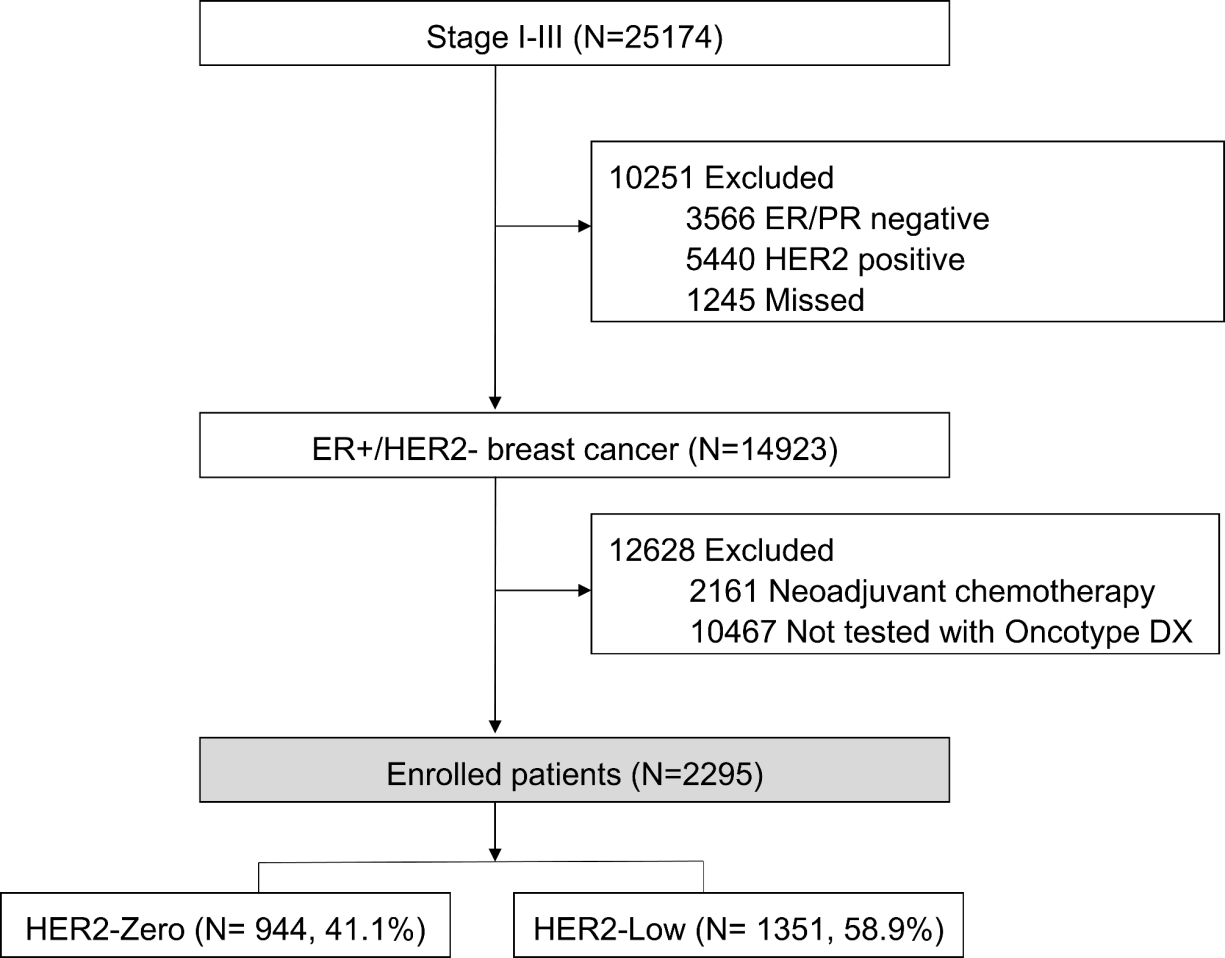
Strobe Diagram of Enrolled Patients. ER indicates estrogen receptor; PR progesterone receptor. ERBB2 + ERBB2-positive; ERBB2- ERBB2-negative

Inclusion criteria for the study required patients to have a diagnosis of invasive breast cancer without metastasis and to have undergone upfront surgery. Additionally, patients needed to have available data on the Oncotype Dx RS, HER2 immunohistochemistry (IHC) score, and, if necessary, silver in situ hybridization (SISH) status. It is important to note that the Oncotype DX test is not covered by the national health insurance in South Korea, and thus, the test was performed selectively. Patients who underwent the test were those who were willing to pay for it privately or for whom the clinician considered the results critical for making treatment decisions. A summary of the study population can be found in Fig. 1.

The decision regarding adjuvant chemotherapy and endocrine therapy, with or without ovarian function suppression, was based on the clinicopathologic characteristics and RS of the tumor and was determined by the treating physician.

### Clinicopathologic characteristics

Clinicopathologic characteristics were obtained from surgical specimens. The hormone receptor status was assessed using the Allred score. HER2-negative was defined as an IHC score of 0, and HER2-low was defined as an IHC score of 1 + or an IHC score of 2 + with a negative SISH result. HER2 IHC and SISH were performed using the Ventana HER2-staining platform, following the ASCO/CAP guidelines in effect at the time of diagnosis. From 2013 to 2017, testing followed the 2013 ASCO/CAP guidelines [18], and beginning in 2018, the updated 2018 ASCO/CAP guidelines were implemented [19]. Other factors such as estrogen receptor (ER), Progesterone receptor (PR) expression, lymphovascular invasion (LVI), Ki-67, histologic grade (HG), and TNM stage were investigated. Ki-67 expression, measured as the labeling index (LI), was assessed through immunohistochemistry (MIB-1; Dako) and reported as a percentage ranging from 0 to 100%, with a high proliferation cutoff of ≥ 20%, based on recommendations from the 2013 St. Gallen International Expert Consensus [20]. 

Menopausal status was determined based on both patient-reported menstrual history and laboratory hormone levels. Patients were classified as postmenopausal if they reported the cessation of menstruation for at least 12 months or if their estradiol (E2) levels were less than 5 pg/mL and their follicle-stimulating hormone (FSH) levels were greater than 30 mIU/mL.

### Recurrence score and gene expression analysis

The 21-gene RS was determined using the surgical specimens at the central laboratory of Genomic Health. RS and gene expression data were obtained from the Oncotype Dx results. Cases with a 21-gene RS > 25 were classified as having high genomic risk, while those with RS ≤ 25 were categorized as low genomic risk for analysis. The quantitative gene expressions of *ESR1*, *PgR*, and *ERBB2* were analyzed using the single-gene score through real-time reverse transcription polymerase chain reaction as indicated in the Oncotype Dx report.

### Statistical analysis

The primary objective of this study was to assess the association between RS and HER2 IHC score. Clinicopathologic characteristics were analyzed, and continuous variables were presented using the student’s t-test, while categorical variables were analyzed using the chi-square test or Fisher’s exact test. One-way ANOVA test was applied to compare means among three group. One-way ANOVA test was utilized to compare means among the three groups. Pairwise comparisons between groups were calculated using the Benjamini-Hochberg method to account for multiple testing [21]. For the univariate analysis, a binary logistic model was used to identify factors associated with high RS. The statistically significant factors identified in the univariate analysis were included in the multivariate analysis to determine the independent determinants of high RS.

Exploratory survival analysis of recurrence-free survival (RFS) and distant recurrence-free survival (DRFS) was conducted using Kaplan-Meier survival curves. RFS and DRFS were defined according to the 2021 Standardized Definitions for Efficacy End Points (STEEP) guidelines [22]. All statistical analyses were performed using SPSS version 26, and a p-value < 0.05 was considered statistically significant for all variables tested.

## Results

### Baseline characteristics

A total of 2295 patients with HR + HER2- patients had available Oncotype Dx scores. Among these patients, 944 (41%) were classified as HER2-zero, and 1351 (59%) were classified as HER2-low. In terms of histologic type, 2027 patients (88.3%) were diagnosed with invasive ductal carcinoma, 184 patients (8%) had invasive lobular carcinoma, and 84 (3.7%) had other histologies. The median age of the overall patient population was 48, with a median age of 48 for HER2-zero patients and 49 for HER2-low patients (Table 1). None of the investigated clinicopathologic characteristics showed a statistically significant difference between HER2-zero and HER2-low patients. The expression of ER and PR by Allred score did not differ significantly between HER2-zero and HER2-low patients.

**Table 1:** Baseline characteristics of patients

Characteristics	HER2 Zero (*n* = 944)	HER2 Low (*n* = 1351)	*p*-value
Age (years)	48 (24–81)	49 (22–79)	0.593
ER (Allred score) *			0.618
Low	8 (0.8%)	9 (0.7%)	
High	936 (99.2%)	1342 (99.3%)	
PR (Allred score) *			0.345
Low	207 (21.9%)	319 (23.6%)	
High	737 (78.1%)	1032 (76.4%)	
Histologic type			
IDC	813 (86%)	1204 (89%)	
ILC	84 (8.9%)	100 (7.4%)	
Others	41 (3.4%)	43 (3.2%)	
Menopausal status			0.930
Premenopausal	606 (64%)	871 (64%)	
Postmenopausal	332 (35%)	473 (35%)	
Unknown	6 (0.6%)	7 (0.1%)	
Tumor Size (n, %)			0.095
≤ 2 cm	628 (66%)	853 (63%)	
> 2 cm	316 (34%)	498 (37%)	
Nodal status (n, %)			0.673
Negative	762 (81%)	1100 (81%)	
Positive	182 (19%)	251 (19%)	
Histologic Grade (n, %)			0.955
1 or 2	848 (90%)	1216 (90%)	
3	96 (10%)	135 (10%)	
Ki-67 (n, %)			0.532
< 20	578 (61%)	846 (63%)	
≥ 20	366 (39%)	505 (37%)	

Unless otherwise noted, values are the number of patients, with percentages in parentheses

* ER/PR Allred score Low: 0–4 / High: 5–8

ER: Estrogen receptor, PR: Progesterone receptor

IDC: Invasive Ductal Carcinoma, ILC: Invasive Lobular Carcinoma

### Association of recurrence score and HER2 expression

**Fig. 2:**
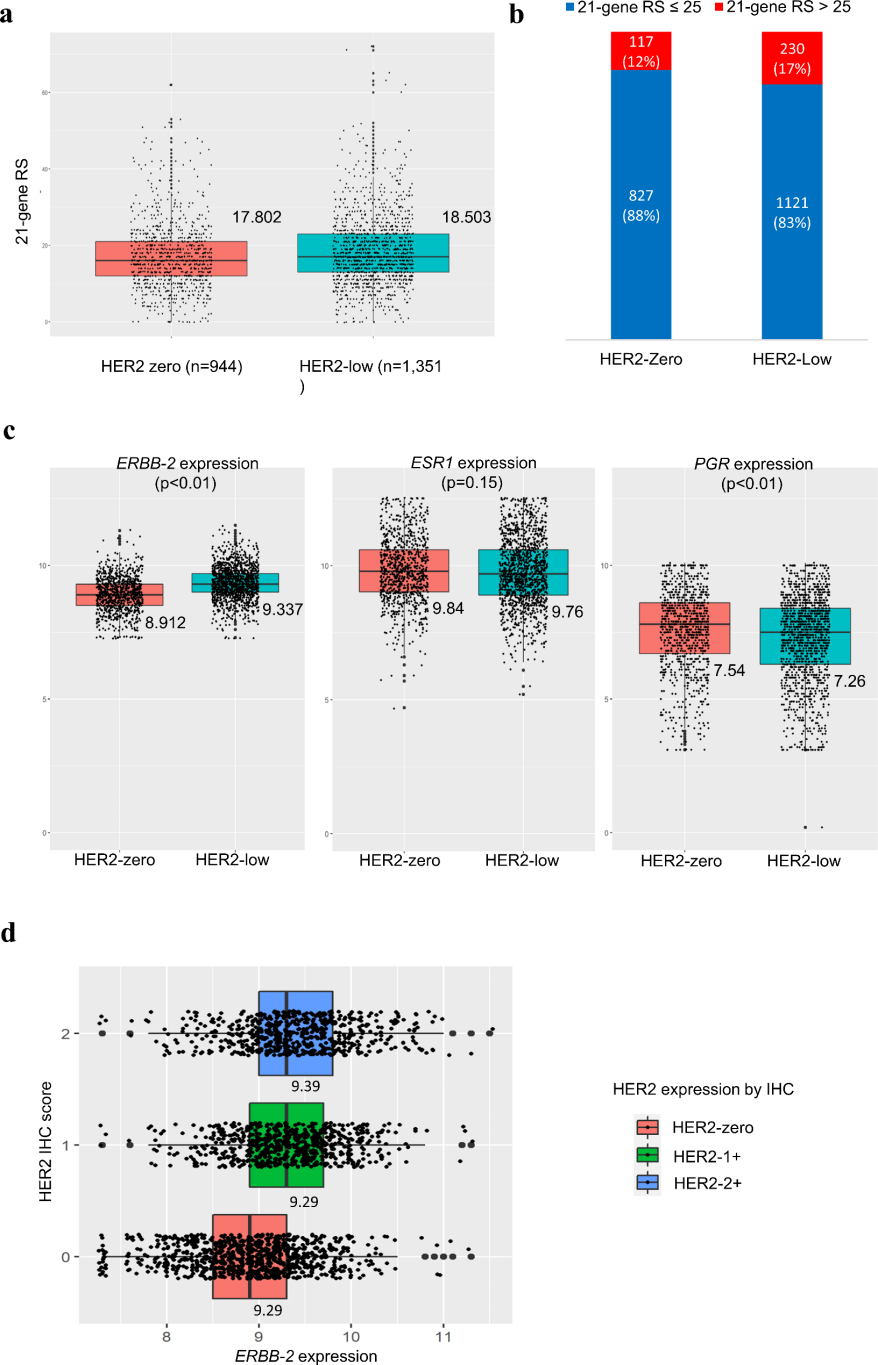
Comparisons of 21-gene recurrence score (RS) and HER2 Status. **(a)** Mean RS is significantly higher in the HER2-low than in the HER2-zero (17.8 vs. 18.5; *P* < 0.001, Student’s T-test) **(b)** Higher proportions of high RS patients in HER2-low patients compared to HER2-zero patients (*P* = 0.002, Chi-square test) **(c)** Gene Expression *ERBB-2*,* ESR1*, and *PGR* were compared between HER2-zero and HER2-low. Significant difference was observed for *ERBB-2* (8.91 vs. 9.33; *P* < 0.01) and *PGR* (7.54 vs. 7.26; *P* < 0.01) expression, while there was no difference in *ESR1* expression (9.84 vs. 9.76; *P* = 0.15) by Student’s T-test. **(d)** Comparison of ERBB-2 expression scores among different IHC scores of HER2 (8.91 in HER2-zero, 9.29 in HER2-1+, 9.39 in HER2-2+; One-way ANOVA test, *p* < 2e-16). Post-hoc analysis was conducted with the Benjamini-Hochberg method. (HER2-zero vs. HER2-1+, *p* < 2e-16; HER2-zero vs. HER2-2+, *p* < 2e-16; HER2-1 + vs. HER2-2+, *p* = 0.0025)

The averages of RS were 17.802 in the HER2-zero group and 18.503 in the HER2-low group (Fig. 2a), demonstrating a statistically significant difference (*p* < 0.01). When comparing the proportion of high RS between the two groups, it was found to be 12.4% (117/944) in the HER2-zero group, while it was 17.0% (230/1351) in the HER2-low group (*p* = 0.002, Fig. 2b).

In addition, mRNA levels of *ESR1*, *PGR*, and *ERBB2* genes were compared according to HER2 IHC status and the results are shown in Fig. 2c. There were nine cases with missing *ESR1* and *PGR* data and 11 cases with missing *ERBB2* data. RNA expression levels showed a statistically significant difference for *ERBB2* and *PGR*, while there was no statistical difference for *ESR1*. The *ERBB2* expression score identified by qRT-PCR was 8.912 in the HER2 zero group and was 9.337 in the HER2-low group (*p* < 0.01). *PGR* expression was 7.54 for HER2-zero and 7.26 for HER2-low (*p* < 0.01), while mean *ESR1* expression was 9.84 and 9.76 respectively (*p* = 0.149). When comparing *ERBB2* gene expression level among the three HER2 group (HER2-zero vs. HER2-1 + vs. HER2-2+), a significant step-wise increase in *ERBB2* gene expression level was observed (Fig. 2d).

### Independent value of Low HER2 for high RS

Next, we addressed an independent value of low HER2 in predicting high RS. ER, PR, and HER2 IHC expression categories, as well as clinicopathologic features such as histologic grade, Ki-67, tumor size, and nodal status, were included in a multivariable analysis using binary logistic regression. HER2-low status demonstrated to be an independent factor for high RS with an odds ratio 1.61 (95% CI: 1.21–2.13, *p* < 0.01), independent of other determinants (Table 2).

**Table 2:** Baseline characteristics of patients

Determinants	Logistic binary regression			
	Univariate analysis	Multivariate analysis		
	*p*-value	Odds Ratio	95% CI	*p*-value
ER (low vs. high)	< 0.01	0.12	0.12 − 0.03	< 0.01
PR (low vs. high)	< 0.01	0.16	0.13–0.22	< 0.01
HER2 (zero vs. low)	< 0.01	1.61	1.21–2.13	< 0.01
Ki-67 (< 20% vs. ≥ 20%)	< 0.01	6.98	5.18–9.39	< 0.01
Histologic grade (1 or 2 vs. 3)	< 0.01	4.08	2.89–5.76	< 0.01
Tumor size (≤ 2 cm vs. < 2 cm)	< 0.01	1.24	0.95–1.63	0.116
Nodal status (Negative vs. Positive)	0.050	0.98	0.68–1.40	0.905

ER: Estrogen receptor, PR: Progesterone receptor, HER2: human epidermal growth factor receptor 2

CI: Confidence Interval

### Survival analysis and chemotherapy-rate

**Fig. 3:**
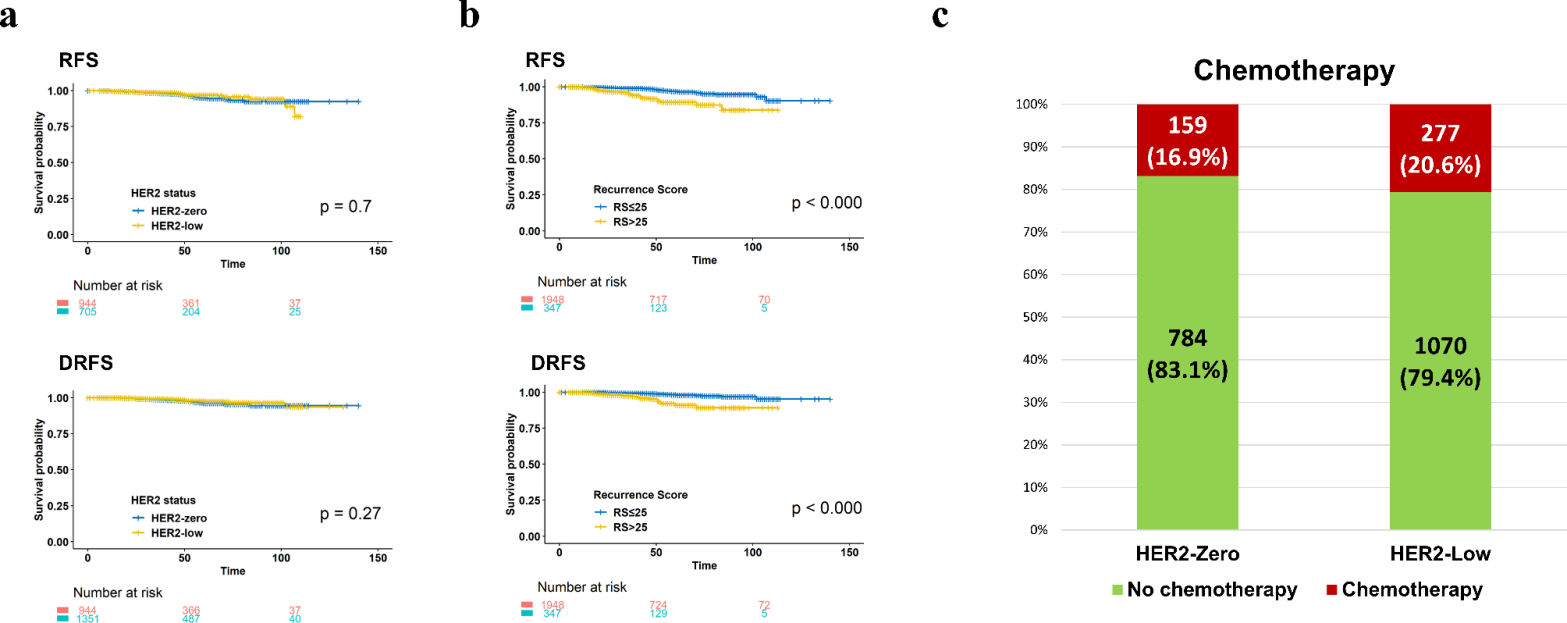
Exploratory Survival Analysis. **a**. Kaplan-Meier Survival analysis for RFS and DRFS showing no difference in survival outcomes between HER2-zero and HER2-low patient groups; **b**. The RFS and DRFS of the RS-low group (RS ≤ 25) were significantly superior to those of the RS-high group (RS > 25); **c**. Ratio of Adjuvant chemotherapy receipt is significantly higher among HER2-low patients compared to HER2-zero patients. (16.9% vs. 20.6%; *P* = 0.026). DRFS, distant recurrence-free survival; RFS, recurrence-free survival

An exploratory survival analysis was conducted to compare the recurrence-free survival (RFS) and distant recurrence-free survival (DRFS) using the Kaplan-Meier survival curve (Fig. 3a). The analysis indicated no statistically significant differences in RFS (log-rank *p* = 0.703) and DRFS (log-rank *p* = 0.274). However, RFS and DRFS appeared to be better for the RS-low group (RS ≤ 25) compared to the RS-high group (RS > 25) (Fig. 3b). When comparing the rate of chemotherapy between two groups, it was observed that a higher proportion of patients in the HER2-low group received adjuvant chemotherapy compared to the HER2-zero group (16.9% in the HER2-low group vs. 10.6% in the HER2-zero group, *p* = 0.026; Fig. 3c).

### Low HER2 and RS according to histology

**Fig. 4:**
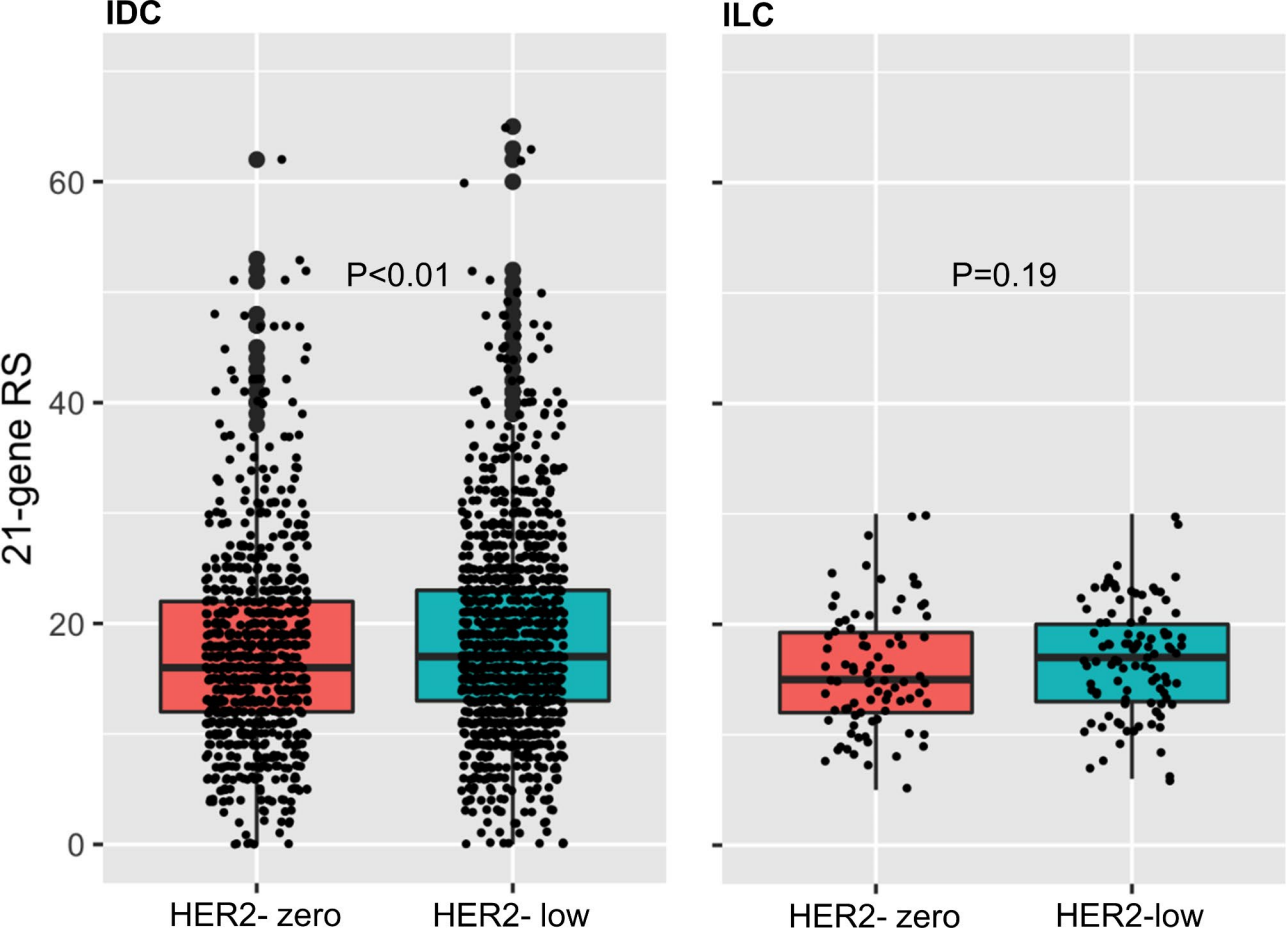
RS Distribution by Histologic Type. Statistically significant higher RS was sustained in IDC (17.4 vs. 18.8; *P* < 0.01) but not in ILC (15.9 vs. 16.9; *P* = 0.19)

The relationship between RS and HER2 expression was further investigated according to histologic types. Among the 2295 patients, three main assessable histologic types were invasive ductal carcinoma (IDC *n* = 2027, 88%), invasive lobular carcinoma (ILC *n* = 184, 8%), and other histology (*n* = 84, 3.7%). For patients diagnosed with IDC, the means of RS for HER2-zero and HER2-low were 17.4 and 18.8, respectively (*p* = 0.001, Fig. 4). However, the average RS for ILC or other histologic types were not different. When RS was categorically divided into genomic risk high and low the high RS rates of HER2-zero and HER2-low were 13.7% versus 18.6% and was significantly different within the IDC subgroup (*p* = 0.003, Table 3).

**Table 3:** Genomic Risk Distribution according to Histology

Genomic Risk Category	HER2 Zero	HER2 Low	*p*-value
Invasive Ductal Carcinoma			0.003
RS ≤ 25	708 (86.3%)	982 (81.4%)	
RS > 25	112 (13.7%)	225 (18.6%)	
Invasive Lobular Carcinoma			0.661
RS ≤ 25	81 (96.5%)	98 (98%)	
RS > 25	3 (3.5%)	2 (2%)	
Other Histology			1.00
RS ≤ 25	38 (95%)	41 (93.2%)	
RS > 25	2 (5%)	3 (6.8%)	

HER2: human epidermal growth factor receptor 2

RS: Recurrence Score

## Discussion

By incorporating the Oncotype Dx RS, we aimed to compare clinical and molecular characteristics of patients with HR + HER2- breast cancer classified as HER2-zero and HER2-low. Notably, this study revealed a slightly higher average RS in the HER2-low group, which was statistically significant. This difference in RS persisted even when the patients were further categorized into high and low genomic risk groups using a threshold of 25. Moreover, HER2-low status was independently identified as a risk factor for high RS. To the best of our knowledge, this study represents one of the largest sample sizes to date in investigating HER2 status using multi-gene assays in HR + HER2- breast cancer [17, 23–25]. However, it is important to acknowledge that this difference in RS was only significant among patients diagnosed with IDC and not among those with ILC.

We observed that this disparity in RS between HER2-zero and HER2-low tumors was associated with increased RNA expression levels of *ERBB2* in the HER2-low tumors. This finding is consistent with a previous study that reported higher expression levels of ERBB2 gene in HER2-low tumors compared to HER2-zero tumors [26]. A notable finding emerged when comparing *ERBB2* gene expression among the three groups based on HER2 status, as it demonstrated a step-wise increase (Fig. 2d). It is not surprising, as previous studies have already established a correlation between HER2 IHC and *ERBB2* gene expression [27–29]. 

Prior research including meta-analysis have suggested data leaning towards how HER2-low has favorable outcomes compared to HER2-zero [23, 30, 31]. For example, a study by Li et al., which included 438 ER + patients with Oncotype DX results, showed that the average RS was higher in HER-2 zero patients compared to HER2-low patients with statistical significance but no difference in the proportion of high RS [30]. Despite the potential contradiction with previous findings, it is important to acknowledge the strength of our study, which lies in the substantial number of patients included (*n* = 2295). The substantial number of patients included in our study enhances the robustness and generalizability of our findings. Additionally, our study differs from others in that we utilized ERBB2 RNA expression levels from Oncotype DX, providing molecular-level insights that differ from traditional HER2 protein-level measurements. These differences may account for the varying results reported in the literature. Furthermore, our relatively young cohort, with a median age of 48 years and a significant proportion of premenopausal patients, offers unique insights into the behavior of HER2-low status in younger populations, making our study distinct from previous research.

In a study conducted by Goldvaser et al., it was observed that high-risk (RS > 25) HER2-low patients demonstrated better survival outcomes, including overall survival, disease-free survival, and distant disease-free survival, compared to high-risk HER2-zero patients in a cohort of 608 patients [23]. In our own study, we found no direct association between higher RS values in HER2-low patients and RFS and DRFS. However, we observed a higher rate of chemotherapy utilization in HER2-low patients, suggesting that chemotherapy may have compensated for the higher genomic risk observed in HER2-low patients. Moreover, it is important to highlight the demographic variations between the aforementioned two studies and our own study. In our study, a significant majority of patients are of Asian ethnicity, and there is a substantial representation of premenopausal individuals. The age distribution in our study is notably lower compared to the other two studies, with an average age of 49 as opposed to 57.9–61 in the other studies [23, 30]. 

This study, while providing valuable insights into HR + HER2-zero/low breast cancer, has certain limitations that need to be taken into consideration. Our study is limited in its inherent design of the retrospective review. Also, we used only the Allred score, as the percentage information for hormone receptor-positive cells is not available for all patients which may affect the granularity of our analysis regarding hormone receptor status. In addition, it is important to note that the Oncotype Dx assay used in this study includes HER2 and GRB7 as part of its 21-gene panel [3]. HER2 and GRB7 have been shown to be co-amplified in breast cancer, and previous studies have suggested that this co-amplification may impact the prognostic value of HER2 status alone [32]. Therefore, the inclusion of these genes in the Oncotype Dx score may have influenced the results of this study. However, the findings of this study are still important, as they provide valuable information on the differences in clinical and molecular characteristics between HER2-zero and HER2-low breast cancer, and suggest that the two groups may have different genomic risk profiles.

In conclusion, our study demonstrates that HER2-low tumors are associated with high Recurrence Scores (RS) in HR + HER2- breast cancer patients, especially in histology of no special type (NST). These findings provide valuable insights into the distinct subtypes of HER2-low in HR + HER2- breast cancer. Further research is needed to explore the prognostic impact of HER2-low expression in HR + HER2- breast cancer.

## Data Availability

The data that support the findings of this study are available from S.G. Ahn asg2004@yuhs.ac, upon reasonable request.

## References

[1] Sørlie T, Perou CM, Tibshirani R, Aas T, Geisler S, Johnsen H, Hastie T, Eisen MB, van de Rijn M, Jeffrey SS et al. Gene expression patterns of breast carcinomas distinguish tumor subclasses with clinical implications. *Proceedings of the National Academy of Sciences* 2001, 98(19):10869–10874. 10.1073/pnas.191367098 PMC58566 11553815

[2] Prat A, Parker JS, Karginova O, Fan C, Livasy C, Herschkowitz JI, He X, Perou CM. <ArticleTitle Language=“En”>Phenotypic and molecular characterization of the claudin-low intrinsic subtype of breast cancer. Breast Cancer Res. 2010;12(5):R68. 20813035 10.1186/bcr2635 PMC3096954

[3] Paik S, Shak S, Tang G, Kim C, Baker J, Cronin M, Baehner FL, Walker MG, Watson D, Park T, et al. A Multigene Assay to Predict Recurrence of Tamoxifen-Treated, Node-Negative Breast Cancer. N Engl J Med. 2004;351(27):2817–26. 15591335 10.1056/NEJMoa041588

[4] Sparano JA, Gray RJ, Makower DF, Pritchard KI, Albain KS, Hayes DF, Geyer CE, Dees EC, Perez EA, Olson JA, et al. Prospective Validation of a 21-Gene Expression Assay in Breast Cancer. N Engl J Med. 2015;373(21):2005–14. 26412349 10.1056/NEJMoa1510764 PMC4701034

[5] Cardoso F, van’t Veer LJ, Bogaerts J, Slaets L, Viale G, Delaloge S, Pierga J-Y, Brain E, Causeret S, DeLorenzi M, et al. 70-Gene Signature as an Aid to Treatment Decisions in Early-Stage Breast Cancer. N Engl J Med. 2016;375(8):717–29. 27557300 10.1056/NEJMoa1602253

[6] Cameron D, Piccart-Gebhart MJ, Gelber RD, Procter M, Goldhirsch A, de Azambuja E, Castro G Jr., Untch M, Smith I, Gianni L, et al. 11 years’ follow-up of trastuzumab after adjuvant chemotherapy in HER2-positive early breast cancer: final analysis of the HERceptin Adjuvant (HERA) trial. Lancet. 2017;389(10075):1195–205. 28215665 10.1016/S0140-6736(16)32616-2 PMC5465633

[7] von Minckwitz G, Procter M, de Azambuja E, Zardavas D, Benyunes M, Viale G, Suter T, Arahmani A, Rouchet N, Clark E, et al. Adjuvant Pertuzumab and Trastuzumab in Early HER2-Positive Breast Cancer. N Engl J Med. 2017;377(2):122–31. 28581356 10.1056/NEJMoa1703643 PMC5538020

[8] Hayes DF. HER2 and Breast Cancer — A Phenomenal Success Story. N Engl J Med. 2019;381(13):1284–6. 31502769 10.1056/NEJMcibr1909386

[9] Piccart-Gebhart MJ, Procter M, Leyland-Jones B, Goldhirsch A, Untch M, Smith I, Gianni L, Baselga J, Bell R, Jackisch C, et al. Trastuzumab after Adjuvant Chemotherapy in HER2-Positive Breast Cancer. N Engl J Med. 2005;353(16):1659–72. 16236737 10.1056/NEJMoa052306

[10] Romond EH, Perez EA, Bryant J, Suman VJ, Geyer CE, Davidson NE, Tan-Chiu E, Martino S, Paik S, Kaufman PA, et al. Trastuzumab plus Adjuvant Chemotherapy for Operable HER2-Positive Breast Cancer. N Engl J Med. 2005;353(16):1673–84. 16236738 10.1056/NEJMoa052122

[11] Nicolò E, Zagami P, Curigliano G. Antibody–drug conjugates in breast cancer: the chemotherapy of the future? Curr Opin Oncol. 2020;32(5):494–502. 32657795 10.1097/CCO.0000000000000656

[12] Modi S, Park H, Murthy RK, Iwata H, Tamura K, Tsurutani J, Moreno-Aspitia A, Doi T, Sagara Y, Redfern C, et al. Antitumor Activity and Safety of Trastuzumab Deruxtecan in Patients With HER2-Low–Expressing Advanced Breast Cancer: Results From a Phase Ib Study. J Clin Oncol. 2020;38(17):1887–96. 32058843 10.1200/JCO.19.02318 PMC7280051

[13] Modi S, Jacot W, Yamashita T, Sohn J, Vidal M, Tokunaga E, Tsurutani J, Ueno NT, Prat A, Chae YS, et al. Trastuzumab Deruxtecan in Previously Treated HER2-Low Advanced Breast Cancer. N Engl J Med. 2022;387(1):9–20. 35665782 10.1056/NEJMoa2203690 PMC10561652

[14] Tarantino P, Hamilton E, Tolaney SM, Cortes J, Morganti S, Ferraro E, Marra A, Viale G, Trapani D, Cardoso F, et al. HER2-Low Breast Cancer: Pathological and Clinical Landscape. J Clin Oncol. 2020;38(17):1951–62. 32330069 10.1200/JCO.19.02488

[15] Denkert C, Seither F, Schneeweiss A, Link T, Blohmer J-U, Just M, Wimberger P, Forberger A, Tesch H, Jackisch C, et al. Clinical and molecular characteristics of HER2-low-positive breast cancer: pooled analysis of individual patient data from four prospective, neoadjuvant clinical trials. Lancet Oncol. 2021;22(8):1151–61. 34252375 10.1016/S1470-2045(21)00301-6

[16] Tarantino P, Curigliano G, Tolaney SM. Navigating the HER2-Low Paradigm in Breast Oncology: New Standards, Future Horizons. Cancer Discov. 2022;12(9):2026–30. 35856622 10.1158/2159-8290.CD-22-0703

[17] Tarantino P, Jin Q, Tayob N, Jeselsohn RM, Schnitt SJ, Vincuilla J, Parker T, Tyekucheva S, Li T, Lin NU. Prognostic and biologic significance of ERBB2-low expression in early-stage breast cancer. JAMA Oncol. 2022;8(8):1177–83. 35737367 10.1001/jamaoncol.2022.2286 PMC9227690

[18] Wolff AC, Hammond MEH, Hicks DG, Dowsett M, McShane LM, Allison KH, Allred DC, Bartlett JMS, Bilous M, Fitzgibbons P, et al. Recommendations for Human Epidermal Growth Factor Receptor 2 Testing in Breast Cancer: American Society of Clinical Oncology/College of American Pathologists Clinical Practice Guideline Update. J Clin Oncol. 2013;31(31):3997–4013. 24101045 10.1200/JCO.2013.50.9984

[19] Wolff AC, Hammond MEH, Allison KH, Harvey BE, Mangu PB, Bartlett JMS, Bilous M, Ellis IO, Fitzgibbons P, Hanna W, et al. Human Epidermal Growth Factor Receptor 2 Testing in Breast Cancer: American Society of Clinical Oncology/College of American Pathologists Clinical Practice Guideline Focused Update. J Clin Oncol. 2018;36(20):2105–22. 29846122 10.1200/JCO.2018.77.8738

[20] Goldhirsch A, Winer EP, Coates AS, Gelber RD, Piccart-Gebhart M, Thürlimann B, Senn HJ, Albain KS, André F, Bergh J, et al. Personalizing the treatment of women with early breast cancer: highlights of the St Gallen International Expert Consensus on the Primary Therapy of Early Breast Cancer 2013. Ann Oncol. 2013;24(9):2206–23. 23917950 10.1093/annonc/mdt303 PMC3755334

[21] Benjamini Y, Hochberg Y. Controlling the False Discovery Rate: A Practical and Powerful Approach to Multiple Testing. J Roy Stat Soc: Ser B (Methodol). 1995;57(1):289–300.

[22] Tolaney SM, Garrett-Mayer E, White J, Blinder VS, Foster JC, Amiri-Kordestani L, Hwang ES, Bliss JM, Rakovitch E, Perlmutter J, et al. Updated Standardized Definitions for Efficacy End Points (STEEP) in Adjuvant Breast Cancer Clinical Trials: STEEP Version 2.0. J Clin Oncol. 2021;39(24):2720–31. 34003702 10.1200/JCO.20.03613 PMC10166345

[23] Mutai R, Barkan T, Moore A, Sarfaty M, Shochat T, Yerushalmi R, Stemmer SM, Goldvaser H. Prognostic impact of HER2-low expression in hormone receptor positive early breast cancer. Breast. 2021;60:62–9. 34481367 10.1016/j.breast.2021.08.016 PMC8414540

[24] Douganiotis G, Kontovinis L, Markopoulou E, Ainali A, Zarampoukas T, Natsiopoulos I, Papazisis K. Prognostic Significance of Low HER2 Expression in Patients With Early Hormone Receptor Positive Breast Cancer. Cancer Diagn Progn. 2022;2(3):316–23. 35530657 10.21873/cdp.10111 PMC9066545

[25] Chen M, Chen W, Liu D, Chen W, Shen K, Wu J, Zhu L. Prognostic values of clinical and molecular features in HER2 low-breast cancer with hormonal receptor overexpression: features of HER2-low breast cancer. Breast Cancer. 2022;29(5):844–53. 35729304 10.1007/s12282-022-01364-y PMC9385837

[26] Schettini F, Chic N, Brasó-Maristany F, Paré L, Pascual T, Conte B, Martínez-Sáez O, Adamo B, Vidal M, Barnadas E, et al. Clinical, pathological, and PAM50 gene expression features of HER2-low breast cancer. NPJ Breast Cancer. 2021;7(1):1. 33397968 10.1038/s41523-020-00208-2 PMC7782714

[27] Du X, Li X-Q, Li L, Xu Y-Y, Feng Y-M. The detection of ESR1/PGR/ERBB2 mRNA levels by RT-QPCR: a better approach for subtyping breast cancer and predicting prognosis. Breast Cancer Res Treat. 2013;138(1):59–67. 23397283 10.1007/s10549-013-2432-2

[28] Noske A, Loibl S, Darb-Esfahani S, Roller M, Kronenwett R, Müller BM, Steffen J, von Toerne C, Wirtz R, Baumann I, et al. Comparison of different approaches for assessment of HER2 expression on protein and mRNA level: prediction of chemotherapy response in the neoadjuvant GeparTrio trial (NCT00544765). Breast Cancer Res Treat. 2011;126(1):109–17. 21190079 10.1007/s10549-010-1316-y

[29] Müller BM, Kronenwett R, Hennig G, Euting H, Weber K, Bohmann K, Weichert W, Altmann G, Roth C, Winzer KJ, et al. Quantitative determination of estrogen receptor, progesterone receptor, and HER2 mRNA in formalin-fixed paraffin-embedded tissue–a new option for predictive biomarker assessment in breast cancer. Diagn Mol Pathol. 2011;20(1):1–10. 21326033 10.1097/PDM.0b013e3181e3630c

[30] Hu Y, Jones D, Zhao W, Tozbikian G, Wesolowski R, Parwani AV, Li Z. Incidence, Clinicopathologic Features, HER2 Fluorescence in Situ Hybridization Profile, and Oncotype DX Results of Human Epidermal Growth Factor Receptor 2-Low Breast Cancers: Experience From a Single Academic Center. Mod Pathol. 2023;36(7):100164. 36967073 10.1016/j.modpat.2023.100164

[31] Ergun Y, Ucar G, Akagunduz B. Comparison of HER2-zero and HER2-low in terms of clinicopathological factors and survival in early-stage breast cancer: A systematic review and meta-analysis. Cancer Treat Rev. 2023;115:102538. 36898351 10.1016/j.ctrv.2023.102538

[32] Stein D, Wu J, Fuqua SA, Roonprapunt C, Yajnik V, D’Eustachio P, Moskow JJ, Buchberg AM, Osborne CK, Margolis B. The SH2 domain protein GRB-7 is co-amplified, overexpressed and in a tight complex with HER2 in breast cancer. EMBO J. 1994;13(6):1331–40. 7907978 10.1002/j.1460-2075.1994.tb06386.x PMC394949

