# Correction: Differentiating HER2-low and HER2-zero tumors with 21-gene multigene assay in 2,295 HR + HER2- breast cancer: a retrospective analysis

**DOI:** 10.1186/s13058-024-01929-z

**Published:** 2024-11-26

**Authors:** Yoonwon Kook, Young-jin Lee, Chihhao Chu, Ji Soo Jang, Seung Ho Baek, Soong June Bae, Yoon Jin Cha, Gyungyup Gong, Joon Jeong, Sae Byul Lee, Sung Gwe Ahn

**Affiliations:** 1 Department of Surgery, Gangnam Severance Hospital, Yonsei University College of Medicine, 712 Eonjuro, Gangnam-gu, Seoul, 06273 Republic of Korea; 2 Institute for Breast Cancer Precision Medicine, Yonsei University College of Medicine, Seoul, Republic of Korea; 3 Department of Surgery, Asan Medical Center, University of Ulsan College of Medicine, Seoul, Republic of Korea; 4 Department of Pathology, Gangnam Severance Hospital, Yonsei University College of Medicine, Seoul, Republic of Korea; 5 Department of Pathology, Asan Medical Center, University of Ulsan College of Medicine, Seoul, Republic of Korea

**Correction to: Kook et al. Breast Cancer Research (2024) 26:154**

10.1186/s13058-024-01911-9

Following the publication of the original article, the authors reported an error in the title due to typesetting mistake. HR + was wrongly typeset as h+.

The title of this Correction is correct, and the original article has been corrected.

